# Supplementary material for: svCapture: efficient and specific detection of very low frequency structural variant junctions by error-minimized capture sequencing
Source: NAR Genom Bioinform. 2023 May 9;5(2):lqad042. doi: 10.1093/nargab/lqad042 (PMC10167630; doi:10.1093/nargab/lqad042)
Supplement: lqad042_Supplemental_Files [file lqad042_supplemental_files.zip › suppl_data/svCapture_supplemental_031123.pdf]

Table S1. Coordinates of target genes and capture regions

Chromosome coordinates are from the GRCh38/hg38 reference genome.

Gene	Chrom	Gene Start	Gene End	Gene Size	Capture Start	Capture End	Capture Size
NEGR1	chr1	71,395,943	72,282,539	887 kb	71,684,317	71,934,317	250 kb
MAGI2	chr7	78,017,055	79,453,667	1.44 Mb	78,460,683	78,710,684	250 kb
PRKG1	chr10	50,990,888	52,298,350	1.31 Mb	51,540,240	51,790,240	250 kb

Table S2. Summary of library sequencing yields and capture enrichment

Yields are expressed as the number of sequenced read pairs and the number of source DNA molecules from which they were derived. Duplex rates are the fraction of on-target proper source molecules detected on both strands. Lines separate sets of libraries handled in the same batch.

Method	Sample	Read Pairs	Molecules	On Target Coverage	Off Target Coverage	Enrichment	Duplex Rate
DuplexSeq	HF1 cell line	292M	69.9M	2,447	5.63	435	48%
DuplexSeq	10% clones	265M	64.9M	2,363	5.28	448	48%
DuplexSeq	1% clones	263M	62.7M	2,290	4.69	489	47%
DuplexSeq	var. % clones	323M	71.5M	2,260	5.50	411	48%
DuplexSeq	? % clones	294M	69M	1,969	5.17	381	48%
DuplexSeq	untreated, #1	314M	81.7M	2,791	7.23	386	48%
DuplexSeq	untreated, #2	289M	92.3M	2,669	7.80	342	49%
DuplexSeq	0.2 uM APH, #1	337M	98M	2,682	8.15	329	49%
DuplexSeq	0.6 uM APH, #1	348M	94.1M	2,344	9.05	259	48%
DuplexSeq	0.6 uM APH, #2	272M	80.4M	2,327	7.07	329	47%
tagmentation	10% clones	113M	6.8M	1,083	0.11	9,669	n.a.
tagmentation	untreated, #1	133M	10.4M	1,188	0.29	4,068	n.a.
tagmentation	0.6 uM APH, #2	120M	7.3M	1,186	0.12	9,561	n.a.

Table S3. Composition of HF1 clone mixtures

HF1 clone	HF1 cell line	10% clones	1% clones	var. % clones	? % clones (blinded)
**GR36**	-	10%	1%	1%	3%
**Scr1-21A**	-	10%	1%	2%	6%
**GR68**	-	10%	1%	3%	20%
**O6A**	-	10%	1%	4%	-
**Scr17A**	-	10%	1%	6%	-
**GR8**	-	10%	1%	8%	-
**E6A**	-	10%	1%	10%	2%
**Scr14A**	-	10%	1%	16%	-
**Hp3-17**	-	10%	1%	20%	1%
**O3A**	-	10%	1%	30%	-
**GR60**	-	-	-	-	4%
**E8A**	-	-	-	-	8%
**Scr1-2A**	-	-	-	-	10%
**GR65**	-	-	-	-	16%
**Scr1-14A**	-	-	-	-	30%
**HF1 cell line**	100%	-	90%	-	-

Table S4. Prior coordinates of known CNVs in clone mixtures

Chromosome coordinates are from the GRCh38/hg38 reference genome, as determined by prior whole genomic microarray analysis.

HF1 Clone	Chrom	CNV Start	CNV End	CNV Size
GR36	chr10	51,616,453	51,648,536	32,083
GR36	chr10	51,628,463	51,647,510	19,047
Scr1-21A	chr1	71,643,114	71,768,387	125,273
Scr1-21A	chr1	71,830,439	72,047,181	216,742
GR68	chr10	51,508,820	51,744,414	235,594
GR68	chr7	78,602,605	78,946,165	343,560
GR68	chr1	71,861,295	71,886,554	25,259
O6A	chr7	78,488,898	78,576,240	87,342
O6A	chr1	71,718,794	72,164,112	445,318
Scr17A	chr10	51,566,939	51,695,648	128,709
Scr17A	chr1	71,902,277	71,958,434	56,157
GR8	chr10	51,576,665	51,676,014	99,349
E6A	chr10	51,684,897	51,750,701	65,804
Hp3-17	chr1	71,781,124	72,066,242	285,118
Scr14A	chr1	71,756,046	71,896,655	140,609
O3A	chr7	78,500,709	78,554,235	53,526
GR60	chr1	71,307,910	71,420,871	112,961
E8A	chr10	51,545,228	51,613,661	68,433
Scr1-2A	chr1	71,797,112	71,857,252	60,140
GR65	chr10	45,876,166	45,892,175	16,009
Scr1-14A	chr1	71,766,605	71,986,149	219,544
Scr1-14A	chr10	51,684,414	51,729,219	44,805
Scr1-14A	chr7	78,478,480	78,640,059	161,579
Scr1-14A	chr7	78,642,674	78,715,891	73,217
Scr1-14A	chr7	78,716,691	78,792,165	75,474

(figures begin on next page)

Figure S1. Structural variant detection and molecule tracking in the svCapture pipeline **(A)** The relevant levels of sequencing redundancy. **(B)** The various ways that source DNA molecules (rows) might fall across a given SV junction (vertical red line) in both unmerged (top) and merged (bottom) read pairs. svCapture tracks the orientation of all alignments relative to split alignments, read-pair gaps, and inner and outer clips and correlates this information with the replicate, *i.e.*, consensus, detection of the sequenced strand(s) of each source molecule. Unique combinations of outer node pairs define source DNA molecules whereas unique combinations of inner node pairs define SV junctions. Depictions are drawn as the pipeline and app operate by showing the DNA spans surrounding the SV junction as they were sequenced, not as they were aligned to the reference genome. In B, proper and anomalous pairing of unmerged reads is considered relative to the gap between the innermost alignments, whereas proper and anomalous merged molecules reflect the absence or presence of a split alignment, respectively.

A

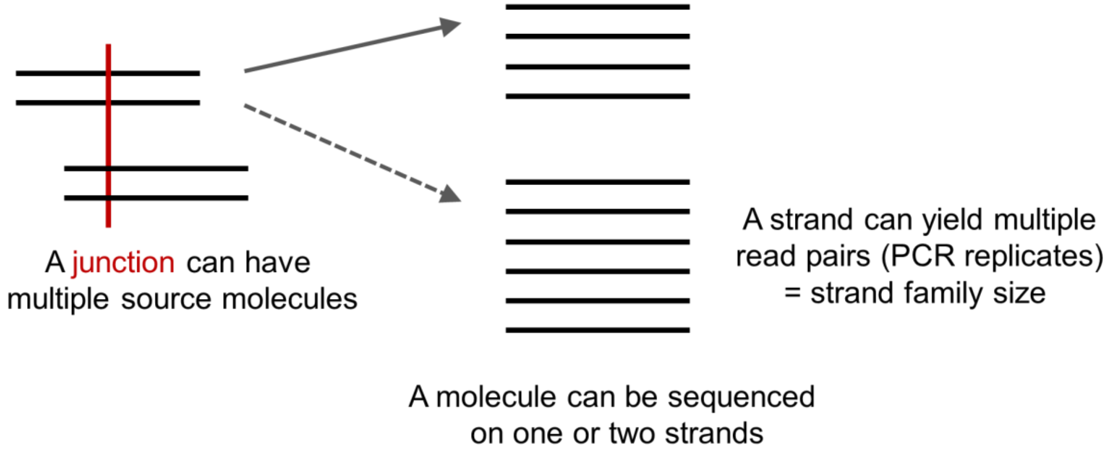

B

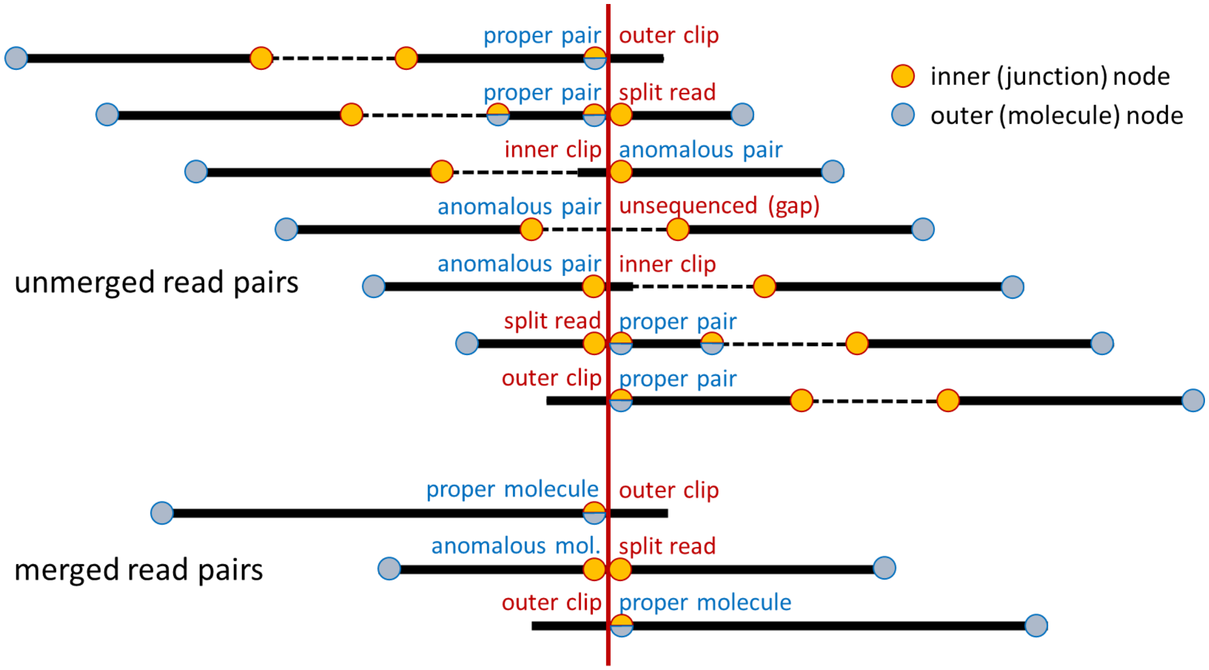

Figure S2. Interpretive guide for plots

(A) Triangular plots show SV endpoint locations relative to the capture target regions, which are highlighted by grey shading. The two endpoints of an SV are inferred by extrapolating diagonally to the x-axis and might be within or adjacent to a capture target or be a translocation between two different capture targets. **(B)** SV junctions are plotted to show the relationship of junction microhomology (x-axis) to SV size (y-axis). Translocations have no size and are arbitrarily plotted at ~1 Mb. Gaussian noise is applied to microhomology lengths to aid in visualization of all data points. Negative microhomology lengths denote *de novo* sequence insertions at junctions. Each dot corresponds to a single SV junction stratified by type (see color legend). Point plot order is randomized to minimize visualization bias due to overplotting.

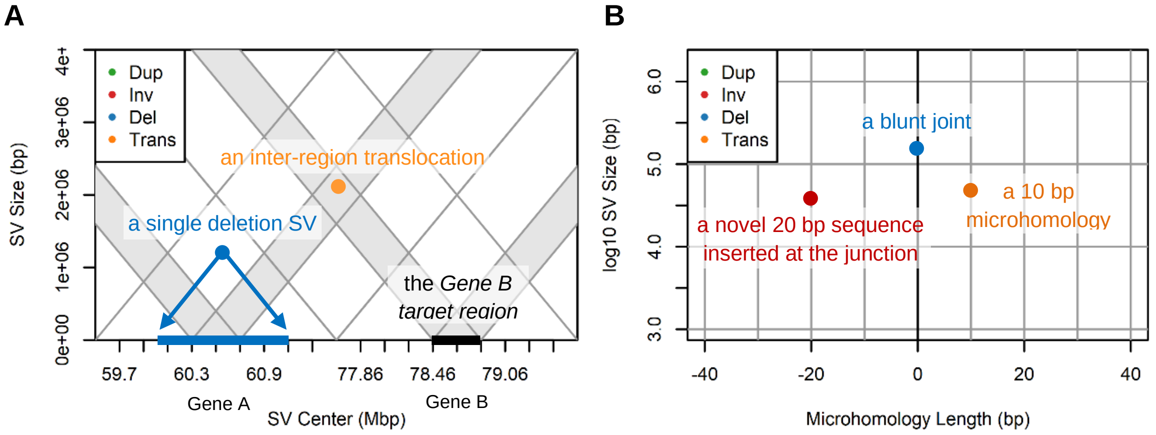

Figure S3. Additional error correction plots from combined DuplexSeq samples
Plots like Figures 2 and 3, now combining all untreated DuplexSeq samples at net 16,789-fold coverage for denser depictions. **(A)** to **(D)** are triangle plots of SV locations analogous to Figure 2A to 2D. **(E)** to **(H)** show SV properties analogous to Figure 3A to 3D.

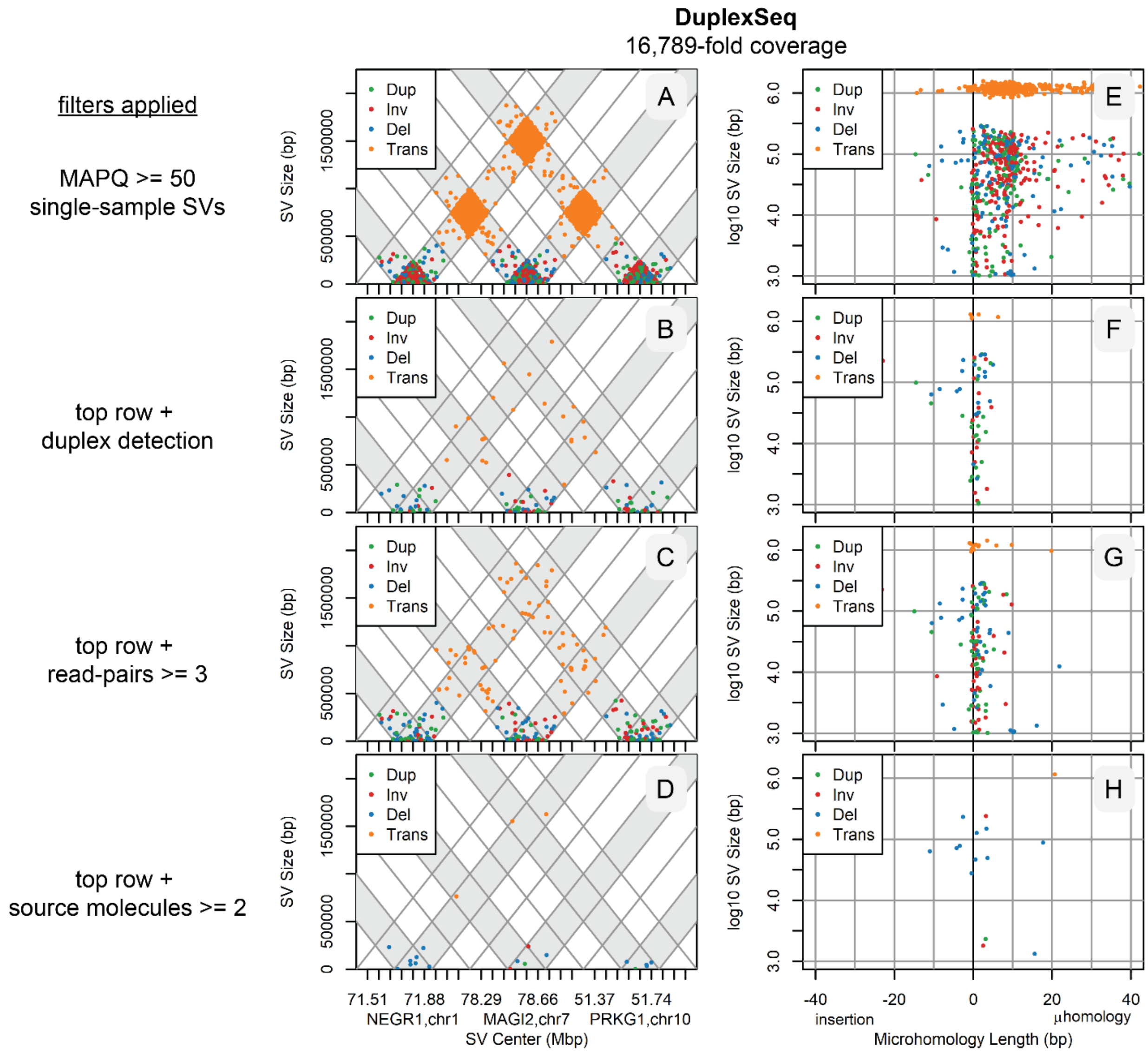

Figure S4. Insert and strand family sizes for DuplexSeq and tagmentation libraries

Figure panels show distributions of **(A)** and **(C)** library insert sizes and **(B)** and **(D)** source DNA molecule strand family sizes for representative **(A)** and **(B)** DuplexSeq and **(C)** and **(D)** Illumina DNA Prep tagmentation libraries. The same untreated HF1 cell input DNA was used for each. See Methods for interpreting "T" (target), "A" (adjacent), and "-" (other) codes. In (C), the abundant class of "TT" proper molecules shows a 10 bp insert size periodicity reflecting the biophysical restrictions of bead-based tagmentation. Strand family size is the number of replicate read pairs corresponding to a given strand of a source DNA molecule. Solid lines are duplex molecules, dashed lines are single strand detections. For each method, "tt" translocation junctions (red) have very small strand family sizes, rarely more than a single read-pair.

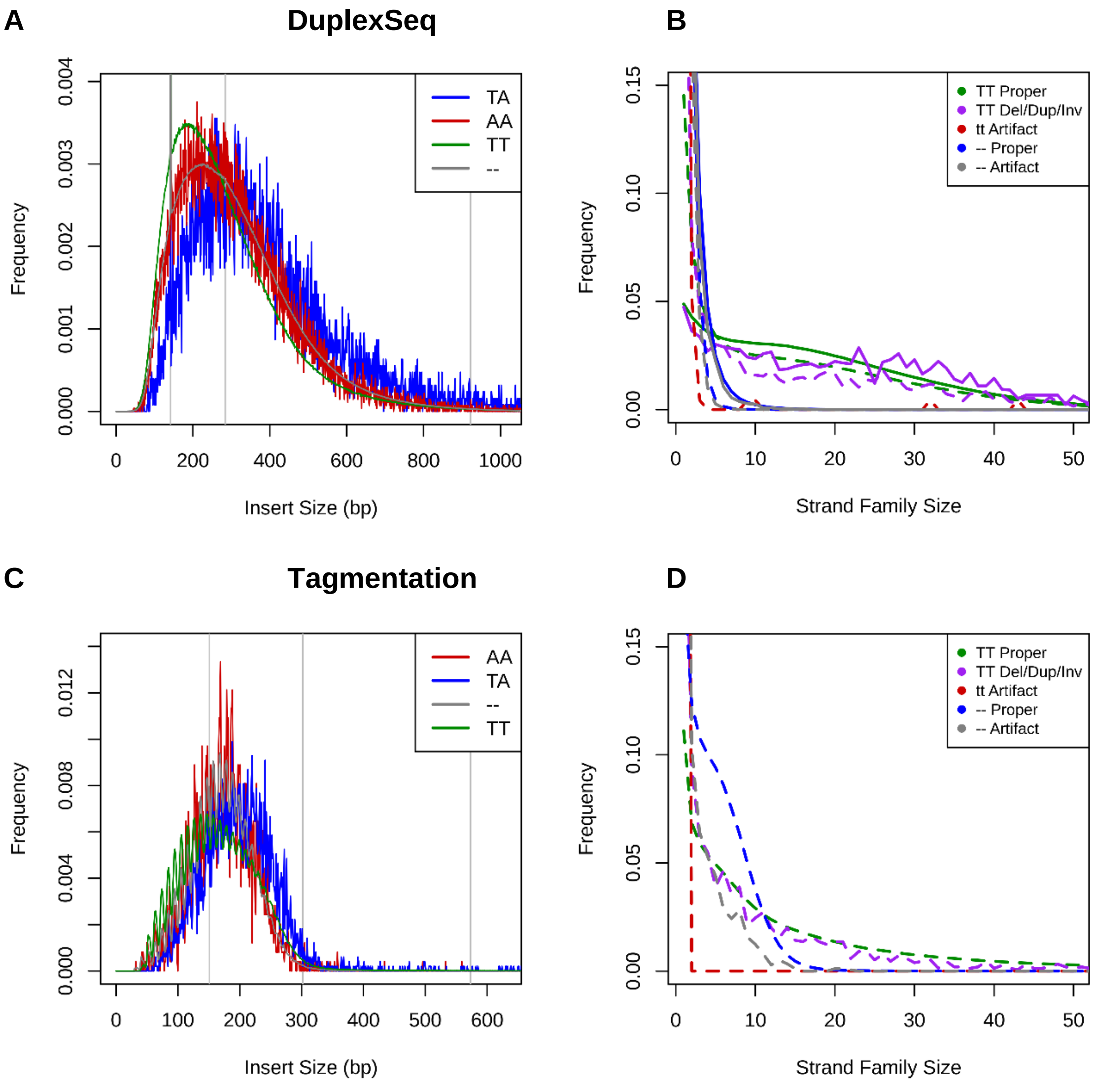

Figure S5. DuplexSeq chimeric PCR artifacts often share endpoints with proper molecules **(A)** Similar to Figure 3A to 3D, now plotting the fraction of fragment ends over all SV-supporting source DNA molecules that matched a proper source molecule in the same library. Most chimeric PCR artifacts at the peak of ~8 to 9 bp microhomologies shared both ends with proper molecules – the presumptive chimeric PCR templates – whereas the cluster of true SVs with a peak of 2 to 4 bp microhomologies did not. Data are from a pool of three DuplexSeq libraries from untreated HF1 cells. Gaussian noise was added to the y-axis values to aid in visualization of all data points, including single-molecule SVs. **(B)** Similar to Figure 2A to 2D for the same data as (A), now requiring that less than 25% of SV molecule ends be shared with a proper molecule.

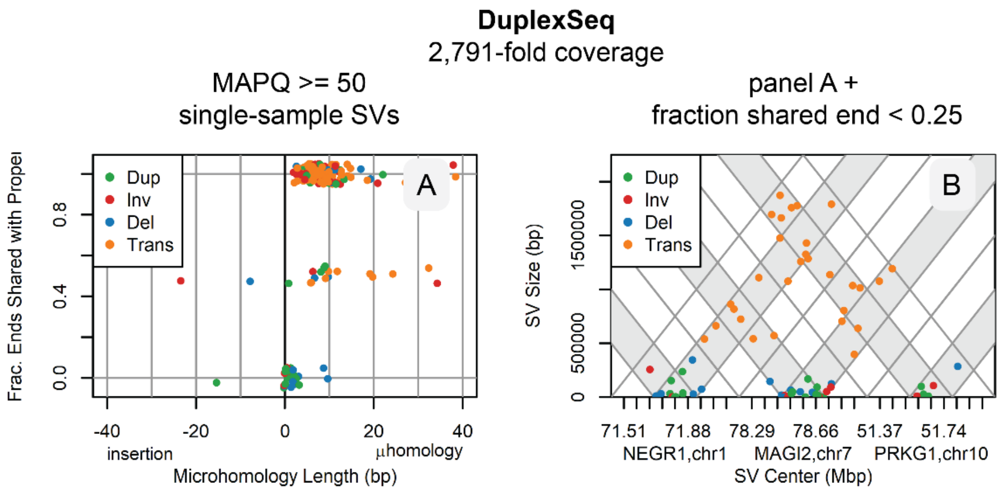

Figure S6. Example molecule evidence for subclonal, mixed-clone SV junctions

Drawings from the svCapture app depict all source molecules supporting example SV junctions. Colors reflect the base values at each position along the sequenced junction (A, green; C, blue; G, brown; T, red; N, black). The top half of each panel shows the alignments to the left side of the junction in bold colors, one alignment per row. The bottom half similarly shows the alignments to the right side of the junction. The clipped, *i.e.*, unaligned, portions are shown in faded colors. The position of the junction is indicated by vertical guidelines, with the internal yellow portions highlighting the microhomology span.

A – 4.5 kb duplication highlighted with a red circle in Figure 5B

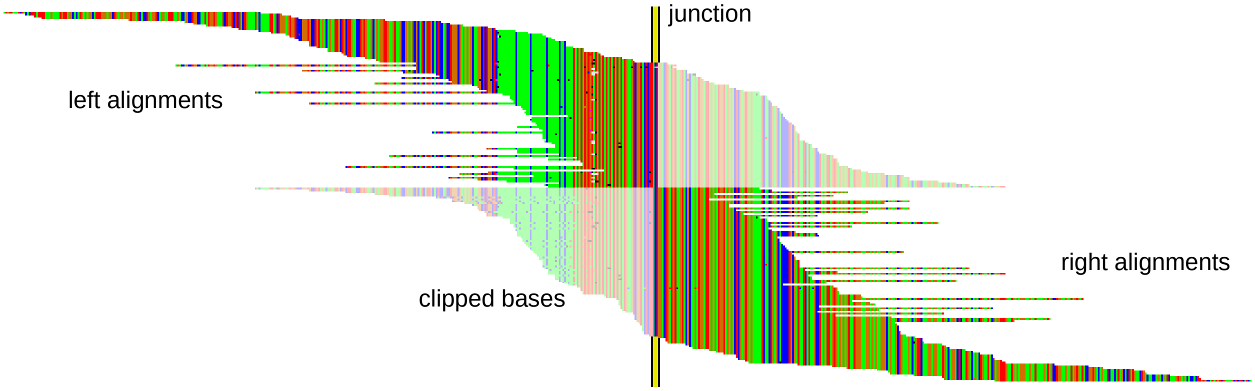

B – 149 kb deletion highlighted with a red circle in Figure 5D (outer edges cropped for clarity)

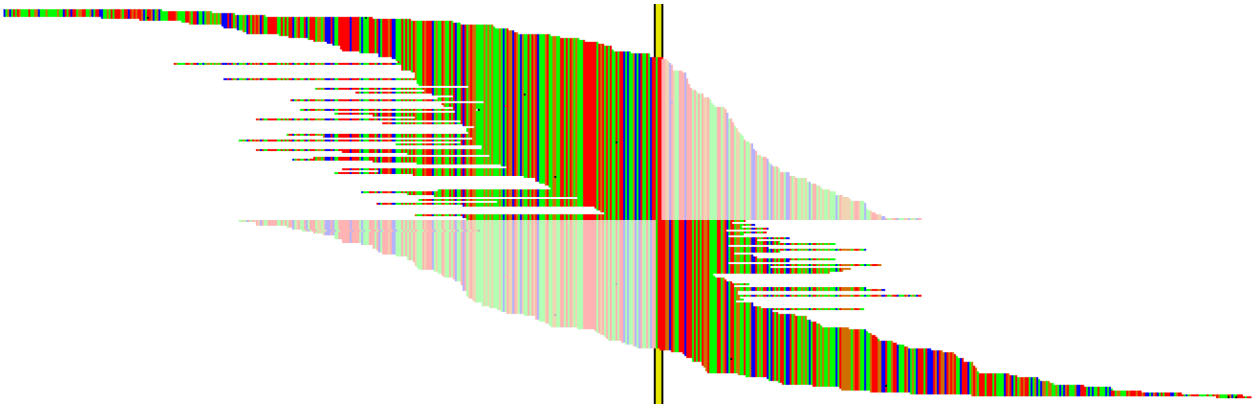

C – 91 kb deletion highlighted with a red circle in Figure 5A

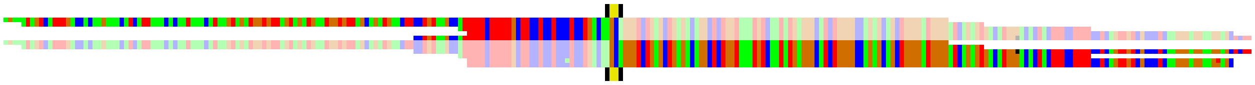

Figure S7. Individual molecule evidence for a *de novo* SNV in an SV junction

The same SV junction depicted in Figure 7A, now showing 10 of the 78 independent source molecule alignments (bottom lines) that sequenced across the variant base (red) as compared to the hg38 reference genome (top line). Lower case bases are the clipped bases in the split alignment. Only a portion of the total read sequences are shown.

```
CTACCTCAGACTCCTGAGTAGCTGGGATTACAGGTGCCCGCCACCATGCCCAGATAATTTTTGTATTTTTAGTAGAAACGGGGTTTCACCACGTTGG
|||||||||||||||||||||||||||||||||||||||||||||||||||||||||X|||||||||||
CTACCTCAGACTCCTGAGTAGCTGGGATTACAGGTGCCCGCCACCATGCCCAGATAAATTTTGTATTTTgaatagatgagatgtgtttttgctagct
CTACCTCAGACTCCTGAGTAGCTGGGATTACAGGTGCCCGCCACCATGCCCAGATAAATTTTGTATTTTgaatagatgagatgtgtttttgctagct
CTACCTCAGACTCCTGAGTAGCTGGGATTACAGGTGCCCGCCACCATGCCCAGATAAATTTTGTATTTTgaatagatgagatgtgtttttgctagct
CTACCTCAGACTCCTGAGTAGCTGGGATTACAGGTGCCCGCCACCATGCCCAGATAAATTTTGTATTTTgaatagatgagatgtgtttttgctagct
CTACCTCAGACTCCTGAGTAGCTGGGATTACAGGTGCCCGCCACCATGCCCAGATAAATTTTGTATTTTgaatagatgagatgtgtttttgctagct
CTACCTCAGACTCCTGAGTAGCTGGGATTACAGGTGCCCGCCACCATGCCCAGATAAATTTTGTATTTTgaatagatgagatgtgtttctgctagct
CTACCTCAGACTCCTGAGTAGCTGGGATTACAGGTGCCCGCCACCATGCCCAGATAAATTTTGTATTTTgaatagatgagatgtgtttttgctagct
CTACCTCAGACTCCTGAGTAGCTGGGATTACAGGTGCCCGCCACCATGCCCAGATAAATTTTGTATTTTgaatagatgagatgtgtttttgctagct
CTACCTCAGACTCCTGAGTAGCTGGGATTACAGGTGCCCGCCACCATGCCCAGATAAATTTTGTATTTTgaatagatgagatgtgtttttgctagct
CTACCTCAGACTCCTGAGTAGCTGGGATTACAGGTGCCCGCCACCATGCCCAGATAAATTTTGTATTTTgaatagatgagatgtgtttttgctagct
Etc.
```
